# European Correspondence

**Published:** 1878-10

**Authors:** R. J. Nunn

**Affiliations:** Paris, France


### EUROPEAN CORRESPONDENCE.

PARIS, FRANCE, 6th September, 1878.

Attending a demonstration on the analysis of urine recently, I was impressed with the clumsiness of the methods employed to determine the albumen. While the sugar is easily determined by the copper precipitated, the uric acid by the nitrogen evolved, the process used for the albumen is so tedious and troublesome that, beyond the bare fact of the presence or absence of albumen, that important element receives but little notice at the hands of the practitioner. The means I have habitually employed is simple and expeditious—so much so that if the albumen is the first element sought for, the analysis will be finished and the result can be ascertained by the time the other researches are completed.

The albumen is precipitated from a measured quantity of filtered urine, in the usual way, and thrown on a filter, the weight of the ash of which has been ascertained. If greater accuracy is desired, the albumen is to be washed with distilled water. As soon as the fluid has passed through, the filter is folded together and pressed between folds of blotting paper, which hasten the process somewhat, but is not essential. The drying can also be hastened by washing with alcohol and ether. The filter, with the alubumen either dry or damp, as the case may be, is next placed in a tube crucible capsule, closed so as to exclude the air, and heated to redness, then cooled and weighed. From the total weight subtract the weight of the vessel and the weight of the ash of the filter; the remainder will be the carbon of the albumen, from which the albumen may be readily calculated.

I spoke, some time since, of the practice now so fashionable among syphilographers, of waiting for the appearance of constitutional symptoms before administering internal remedies—a practice which rests a good deal, perhaps, upon the theories of Ricord. A great part of the measures adopted by physicians, are used with the intention of averting anticipated evils which might or might not occur. Quinine is often given, not for the chill a patient had yesterday, nor for the fever he had this morning, but to prevent the symptoms anticipated to-morrow or next day, and the removal of a diseased eye is advised to prevent a possible injury to the sound one, yet who can say positively that either of these contingencies would occur? The expectant syphilographer asks defiantly if a case can be pointed out in which the early use of mercury prevented secondary symptoms. He asks an unanswerable question, and plumes himself accordingly, but this much can be said in reply: Every physician of experience has seen cases of chancre in which mercury was given, and in which secondary symptoms did not occur during the life of the patient. Certainly the practice seems to me not unlike deferring vaccination until the patient is attacked with small-pox. And, again, it is past comprehension to understand how a remedy should be unable to destroy a few germs, but should become all-powerful when these have multiplied indefinitely. But to a case in point. A patient appears before a physician having upon the outside of the penis a small scab having all the appearance of a herpes. There was no swelling of glands, nor other symptoms of syphilis; the physician diagnosed a case of herpes, and advised treatment accordingly, but added that even if it was a case of syphilis, he would advise waiting until constitutional symptoms would manifest themselves before giving minerals. Now, what was the result? The case turned out to be syphilis, with severe constitutional symptoms.

Pathologists agree that it is impossible to distinguish between a herpes and some of the earlier forms of syphilis; but I would go a step further, and indicate the existence of a communicable syphilitic herpes, which may vary its course according to the diathesis of the individual affected; but which, in its early stages, is not distinguishable from non-syphilitic herpes.

I saw the case in question, and while stating candidly the fact that an accurate diagnosis was impossible, I was inclined to look upon the case as sufficiently suspicious to warrant constitutional treatment if he had been my patient; the physician subsequently consulted took, however, a different view of the case and its treatment, and as I am not engaged in practice here, I had no tangible objections to offer—it was simply a question of opinion and experience.

But here comes in the question of diathesis, that undefinable, ever-varying condition of the system which causes one person to be more susceptible to a particular disease than another, and changes the degree of susceptibility at different periods of the life of the same individual. It is this uncertain element, this ever-changing factor, which prevents the application of logical rules to medicine with anything like certainty of results; ignoring this, the practitioner becomes a mere routinist, the medical teacher, a dogmatist. It follows that the opinions based upon the observations made in the practice of one or a dozen physicians may be absolutely valueless when applied to the patients attended by another. It follows that all these medical authorities should be regarded as suggestive only, and not as indicating a rigid rule of practice. Among the curiosities of syphilis, recently brought to my notice, were three cases inoculated by the same woman on the same night, treated in the same hospital by the same physician and similar medication (mercurial); of these, the first had secondary symptoms, but was cured (?) without accident; the second had no other symptom than the chancre, while the third died.

At the meeting of the British Medical Association last year, I was forcibly struck with the tendency of teachers to become dogmatists. A paper had just been read on the introduction of the hand into a subinvoluted uterus some days after delivery to extract an adherent piece of placenta which was causing hemorrhage. During the discussion which followed, a prominent professor stated it to be a most dangerous practice to introduce the hand into the uterus after delivery, modestly adding, "among the class of patients attended by himself." Now, it happens that my experience is altogether different, for among those patients attended by me, it has always been my rule—not invariable, of course—to introduce the hand as soon as possible after delivery of the child, and then retain it until it was expelled with the secundines. I thus avoid irregular contractions and retained clots, and consequently diminish the after-pains to a considerable extent; furthermore, the introduction of the hand at this time gives little or no pain to the patient, as the external parts have not yet contracted or tumefied. I only mention this to show that what might be proved by experience—after all the only real medical teacher—to be good practice in Savannah, might not suit in the crowded population of an English city. The surgery, which might be very successful on the dry table lands of Mexico, might be most disastrous in the damp climate of England.

In the treatment of stricture of the urethra, I find practice pretty well divided, some rupturing, some incising, some galvanizing and some gradually dilating. On the whole, I think the two latter methods have the preference in private practice.

In the gynecological clinic of M. Trippier, the other day, he drew attention to the extraordinary prevalence of hepatic diathesis among the Russians and scrofula among the Israelites. Certainly, I think this assertion will not be borne out by observation in our section of country, and I am quite sure that my personal experience is quite opposed to this, as far, at least, as the Hebrews are concerned. M. Trippier appears to look upon diathesis as the great predisposing cause to uterine complications, while M. Chum advocates the nervous origin of these disorders. Of course the result is a wide diversity of practice, the practical results of which must be looked for in the works of the observers in question, as, of course, the comparative success must be the result of years of observation.

Among the curiosities of the exhibition is the new metal Gallium, discovered by M. Lecoq de Boisbaudran, but of its properties I have no accurate knowledge.

As an excipient for intra-uterine medication, I find soup is a good deal employed, but I do not think it is as good as the calloids, (the vegetable gums, or the gelatines,) which can be made of any consistency desired by the addition of glycerine, and hence the practitioner has it in his power to keep the parts bathed, as it were, with medicament, for as long or as short an interval as he chooses. For years I have employed this method of applying remedies to the uterus, vagina, rectum, etc., with satisfactory results, and I would suggest the medicated calloids as an excellent mode of internal medication.

It is often desirable to apply remedies to the os for some hours at a time without permitting them to come in contact with the vaginal walls. This end can, to a great extent, be attaind by the use of a thin india-rubber sack, attached to an india-rubber ring. The ring encloses the *col* which protrudes, within the sack in which is placed the dressing desired.

A modification in the canulas used for vaginal injection, upon which I always insist, is to have the end hole closed entirely, and the direction of the other changed so as to give a reversed current, discharging towards the exterior instead of the interior. This little change was the result of three or four severe cases of colic and subsequent inflammation, occasioned by the entrance of the injection into the uterine cavity.

One of the most instructive and entertaining of Parisian cliniques, is that of Dr. Ch. Fauvel, for diseases of the larynx, whose "Practical Treatise on Diseases of the Larynx, preceded by a complete Treatise on the Laryngoscope," will probably be the most complete and exhaustive work on the subject extant. The first volume, of 900 pages, is already published, and is in itself a complete treatise, embracing the history of laryngoscopy, a description of the necessary apparatus, methods of examination and treatment, which take up the first part—rather more than one-sixth the volume—while the remainder is occupied with the discussion of laryngeal tumors, polypi, cancers and catarrh.

The work contains notices of 300 cases of tumors of the larynx, which have come under the author's notice. Of these, 233 were operated on, and the results obtained were 180 cures, 48 improved, and 5 unbenefitted. The value of laryngoscopy may be estimated from these figures. Not many years ago death by suffocation, or tracheotomy, would have been the end of these cases.

The chapters on cancer are a valuable addition to medical literature, as heretofore there are but few cases recorded of primary cancer of the larynx. The left side is more frequently the seat of this disease than the right, in the proportion of 26 to 7, and from forty years of age to seventy, is the period most liable to be attacked. Thirty-four men were affected with cancer of the larynx, and but three women, which, Dr. Fauvel suggests, may be owing to the localization of the disease in the mamma or uterus of the latter.

An important point in the diagnostic differences between cancer and laryngeal phthisis is, that in the former, tbe voice is seldom completely destroyed, while in the latter articulation is often absolutely impossible; and cancerous patients are always submitted to an anti-syphilistic treatment, so great is the difficulty of destinguishing between laryngeal cancer and syphilis. The danger of neglecting laryngeal growths—although apparently stationary, is pointed out as leading to sudden attacks of suffocation, from their rapid development. The operation of thyrotomy is strongly condemned as being useless and dangerous from hemorrhage; in one case thirty-eight ligatures had to be employed. A point of practical importance is well made, which is, that all solutions should be applied during expiration, thus avoiding spasm.

Since the invention of the laryngoscope no such work as this has been published on the subject, and certainly no laryngoscopist, who desires to be fully informed on the subject with which he is dealing, can afford to be without it.

To the casual medical traveler, Paris, for the past month or two, has been most uninteresting. All the "cliniques" at the hospitals have been suspended for the vacation; and the professors are mostly rusticating; so that unless for persons, who like myself, are engaged in making special observations and investigations at private cliniqes, and with private professors, there is really nothing to be seen; this is the case even in surgery; for all operations that can possibly be deferred will be put off until the course of winter teaching begins.

R. J. Nunn.

